# Examination of Genetic Variants Revealed from a Rat Model of Brain Ischemia in Patients with Ischemic Stroke: A Pilot Study

**DOI:** 10.3390/genes12121938

**Published:** 2021-11-30

**Authors:** Andrey V. Khrunin, Gennady V. Khvorykh, Alexandra V. Rozhkova, Evgeniya A. Koltsova, Elizaveta A. Petrova, Ekaterina I. Kimelfeld, Svetlana A. Limborska

**Affiliations:** 1 Institute of Molecular Genetics of National Research Center “Kurchatov Institute”, Kurchatov Sq. 2, 123182 Moscow, Russia; khvorykh@img.msk.ru (G.V.K.); avrojkova@img.msk.ru (A.V.R.); limbor@img.msk.ru (S.A.L.); 2 Department of Neurology, Neurosurgery and Medical Genetics, Pirogov Russian National Research Medical University, 117997 Moscow, Russia; koltsovaevgenia@rambler.ru (E.A.K.); 6332011@mail.ru (E.A.P.); ekovita@mail.ru (E.I.K.)

**Keywords:** single nucleotide polymorphisms, ischemic stroke, ischemic stroke outcome, rat model of brain ischemia

## Abstract

Although there has been great progress in understanding the genetic bases of ischemic stroke (IS), many of its aspects remain underexplored. These include the genetics of outcomes, as well as problems with the identification of real causative loci and their functional annotations. Therefore, analysis of the results obtained from animal models of brain ischemia could be helpful. We have developed a bioinformatic approach exploring single nucleotide polymorphisms (SNPs) in human orthologues of rat genes expressed differentially under conditions of induced brain ischemia. Using this approach, we identified and analyzed nine SNPs in 553 Russian individuals (331 patients with IS and 222 controls). We explored the association of SNPs with both IS outcomes and with the risk of IS. SNP rs66782529 (*LGALS3*) was associated with negative IS outcomes (*p* = 0.048). SNPs rs62278647 and rs2316710 (*PTX3*) were associated significantly with IS (*p* = 0.000029 and *p* = 0.0025, respectively). These correlations for rs62278647 and rs2316710 were found only in women, which suggests a sex-specific association of the *PTX3* polymorphism. Thus, this research not only reveals some new genetic associations with IS and its outcomes but also shows how exploring variations in genes from a rat model of brain ischemia can be of use in searching for human genetic markers of this disorder.

## 1. Introduction

Stroke, including ischemic stroke (IS), is a multifactorial disease and one of the main causes of morbidity and mortality worldwide [1]. Several issues are known to influence the risk of IS, and genetic factors can make essential contributions to an individual’s susceptibility. Depending on the subtype of IS, the estimated heritability has ranged from 16.1% for small vessel disease to 32.6% and 40.3% for cardioembolic and large vessel disease, respectively [2]. The last largest genome-wide association (GWA) study revealed 22 new stroke risk loci, bringing the number of known stroke-associated loci to 32, comprising 149 genes [3]. The outcomes of IS are also influenced by different factors, including genetic ones [4]. However, in contrast to IS itself, our knowledge of the genetics of poststroke outcome is very limited. Only a few GWA studies have been performed for this, and several loci were found to be associated [5,6,7]. Because these studies proved effective in revealing trait-associated loci, further such studies are expected. However, GWA studies suffer from some limitations. For example, they have poor precision in identifying real causal variants or genes caused by the linkage disequilibrium-based architecture of DNA arrays used, which has hindered the development of biological insights and clinical translation of GWA findings, and requires functional validation of each gene association discovered [8]. Ultimately, this deficiency highlights the need for the development of new approaches that can delineate causal genetic variants and biological mechanisms underlying the associations observed with IS. They can be realized in enhanced downstream characterization of identified loci through the involvement of different ‘omics’ data and advanced analytic techniques, or in direct functional dissection of particular variants and genes [8,9,10]. 

In parallel, the genetics of IS have also been studied intensively in animal models [11,12]. Some of the results obtained in rodent models of stroke have been confirmed or correlated with the results of association studies in humans, including GWA-assessed outcomes after IS [5], but their translation remains generally low. This is because of substantial physiological differences between animal models and humans, and the inadequacy of animal models of stroke [12]. Although the creation of more perfect animal models of the diseases has been declared as one method of improving the translation, relevant results can be obtained through the development of approaches based on careful selection and cross-species multi-omic data validation [13,14]. Following similar principles, we have recently developed a bioinformatics protocol to search for genomic markers that could affect IS outcome [15]. The protocol bears certain similarities with the method of Pasterkamp et al. [16] that was used for the human validation of atherosclerosis-causing genes studied in knockout and transgenic mice. Our method differs in approaches to the selection of both potential candidate genes, which are human orthologues of rat genes that have demonstrated the most significant changes in their expression in the brain as a response to temporal artery occlusion, and SNPs, including functionally important SNPs. Here, we present the results of an association study of nine genetic variants which have been identified with this protocol. Both outcomes and the risk of IS were tested.

## 2. Materials and Methods

### 2.1. Study Subjects

The association study was carried out on a subgroup of patients with IS admitted to the Neurologic Department of Moscow City Clinical Hospital No. 31 during 2009–2011. Detailed procedures of patient enrollment and data collection have been described previously [17]. Briefly, the study group comprised both men and women aged ≥55 years, diagnosed with their first IS, and taken to hospital at the acute stage of the disease. Patients with transitory ischemic attacks, recurrent stroke, hemorrhagic stroke, acute myocardial infarction, decompensated heart failure, and other severe accompanying conditions, including autoimmune and infectious diseases, were not recruited into the study. The stroke subtypes were defined according to the Trial of Org 10172 in Acute Stroke Treatment criteria [18]. To assess stroke severity, the National Institutes of Health stroke scale was used [19]. The functional outcome after stroke was graded using a modified Rankin scale (mRS) [20]. Severity and outcome were assessed twice, at days 1 and 14 after the stroke event. To obtain a group with homogeneous ancestry, only self-described Russian patients were included. In all, 331 patients (166 males and 165 females; mean age 71.68 ± 8.69) with IS were enrolled. The control group included 222 healthy individuals (109 males and 113 females; mean age 70.20 ± 9.63) with Russian ancestry from regions of Central European Russia. All subjects provided written informed consent for participation.

### 2.2. Selection and Genotyping of Markers

DNA was isolated from peripheral blood cells by proteinase K treatment, followed by extraction with phenol–chloroform [21]. The principles of the selection of polymorphic markers analyzed in the study were described in our recently developed protocol [15]. They included the following main steps: (1) Selection of rat genes that showed the most significant changes in their expression in the brain in response to temporal artery occlusion; (2) Identification of the human orthologues of these rat genes; (3) Identification of SNPs within human genes and revealing among them any informative SNPs with a high degree of linkage disequilibrium (tagSNPs). These require whole genome sequence data from an appropriate population (in our study, it was CEU population from 1000 Genomes Project); (4) Annotation and identification of functionally important tagSNPs that affect the expression of the genes studied, namely expressed quantitative trait loci (eQTLs) (GTEx Analysis Release V8 was used to annotate the tagSNPs). The annotation resulted in a dramatic drop in the number of candidate genes, discarding those with low potentials. In evaluating the tagSNPs, we first selected the SNPs associated with changes in the expression of corresponding genes in human brain tissues. There were eight such SNPs in five genes (*PTX3, RGS9, EMP1, CD14*, and *LGALS3*) (Table 1). An additional SNP (locus rs1491961 in *CCR1*) was included in the analysis based on its prominent eQTL-related statistics. It had the greatest absolute *p*-value and normalized effect size (10^–47^ and –0.40, respectively) and its functional effect was associated with expression in whole blood.

The selected tag SNPs were genotyped in the current study with the use of TaqMan real-time PCR assays. Primers and probes for allele discrimination were designed using Beacon Designer software v. 7.91 (PREMIER Biosoft International, San Francisco, CA, USA) and were synthesized by DNA-Synthesis Company (Moscow, Russia) (Table 1). Polymerase chain reaction (PCR) was performed in water solution with a final volume of 25 μL, containing 2.5 μL of 10× PCR buffer (Syntol, Moscow, Russia), 2.5 mM MgCl_2_ (Sileks, Moscow, Russia), 250 μM each of deoxyribonucleoside triphosphates (Thermo Fisher Scientific Baltics UAB, Vilnius, Lithuania), 10 pM each of primers (DNA-Synthesis, Moscow, Russia), 5 pM of FAM- and VIC-specific TaqMan probes (DNA-Synthesis, Moscow, Russia), 1.25 units of SynTaq DNA polymerase (Syntol, Moscow, Russia) and 50 ng of genomic DNA. Thermal cycling and fluorescence intensity measurement were conducted using a StepOnePlus Real-Time PCR System (Applied Biosystems, Waltham, MA, USA). The samples were initially incubated for 10 min at 95 °C for activation of the SynTaq DNA polymerase and then cycled 40 times at 95 °C for 15 s, followed by 54–62 °C for 45 s (Table 1). All the genotypes obtained are presented in Supplementary Table S1.

### 2.3. Statistical Analysis

The aim of this study was to explore the associations between genetic variants and IS outcomes as well as the risk of developing the disease. The effects of SNPs on stroke outcome were assessed through their associations with mRS scores. Scores were analyzed as follows: (1) mRS scores of 0–2 vs. scores of 3–6 (Study 1); (2) mRS scores of 0–3 vs. scores of 4–6 (Study 2); and (3) changes in mRS scores from days 1 to 14 following IS (Study 3). The first two studies differed according to the level of help required by the patients in everyday life (mRS scores of 0–2 vs. scores of 3–6; functional recovery group 1 and nonfunctional recovery group 1); the ability of patients to walk (mRS scores of 0–3 vs. mRS scores of 4–6; functional recovery group 2 and nonfunctional recovery group 2); and were based on the mRS scores assessed at day 14. Study 3 was conducted to evaluate the differences in direction of poststroke outcomes. These were assessed through the calculation of individual changes (∆ values) in mRS scores given to patients at days 1 and 14 as ∆mRS = mRS1–mRS14 and defined as positive (∆mRS > 0), negative (∆mRS < 0), or stable (∆mRS = 0). To access the relationships between the genotypes and variables, the genotypic *χ*^2^ test was used. When there was a need to clarify the contribution of particular genotypes, dominant, recessive, and overdominant genetic models were additionally applied. To find independent variables, multiple linear regression was used. The results were adjusted for age and sex [3,5,22]. The significance of associations was set at *p* < 0.0056, which was calculated as the nominal value (*p* < 0.05) divided by the total number of SNPs tested (n = 9). Statistical analyses were performed using Statistica software v. 8.0 (StatSoft, Inc., Tulsa, OK, USA), R package AssocTests [23], and Haploview software v. 4.2 [24].

## 3. Results

Nine SNPs were genotyped in the current study. The frequencies of genotypes according to the groups studied are listed in Table 2 and Table 3. The distributions of all genotypes in control samples did not deviate from expected values under Hardy–Weinberg equilibrium.

Of 331 IS patients analyzed in Study 1, 116 were classified as the functional recovery group (mRS scores of 0–2) and 214 were nonfunctional recovery group (mRS scores of 3–6) (one patient was excluded from the analysis because he died at day 8 after IS). In Study 2, the functional recovery group included 175 patients (mRS scores of 0–3) while the nonfunctional recovery group comprised 155 patients (mRS scores of 4–6). One hundred and fifty-five patients demonstrated positive dynamics in changes to their mRS scores (∆mRS > 0); negative changes were detected in 67 patients (∆mRS < 0); and 108 patients were stable (∆mRS = 0) (Table 2). No substantial differences in genotype distributions were found between groups in Studies 1 and 2. At the same time, analysis of the genotype distributions observed in Study 3 revealed one nominally significant SNP (*p* < 0.05), namely rs66782529 (Table 2). Additional pairwise comparisons suggested that this association was caused by a distinct genotype pattern in the group of patients with negative ΔmRS values, particularly by a higher frequency of genotype CC in this group compared to two others. The association was the most important in the comparison of groups with negative and stable ΔmRS values where rs66782529 was an independent predictor of IS outcome (*p* = 0.0047 compared to *p* = 0.9235 and *p* = 0.3071 for sex and age, respectively).

Because the genetics of IS are not yet clearly understood, and experiments in animal models cannot be absolutely relevant to humans, the SNPs were also tested for association with the risk of IS. Two SNPs showed significant associations with the disease: rs62278647 and rs2316710 (Table 3). Both SNPs belonged to the *PTX3* gene and were in strong LD in our sample (*r*^2^ = 0.82). Combining information on genotype distribution in terms of the genetic models of inheritance (dominant, recessive, and overdominant) showed the most pronounced association with a heterozygous genotype AT in the patients (*p* = 0.000027). In the case of SNP rs2316710, such prevalence was also observed but it was not significant (*p* = 0.0095) in that it corresponded to the results of regression analysis where SNP rs62278647 was the only independent predictor of IS disease. Additional comparisons performed separately in male and female cohorts showed that the associations found were significant only in the females (*p* = 0.000057 and *p* = 0.000765 for rs62278647 and rs2316710, respectively). Both associations remained significant even after correction for the number of two additional comparisons performed (*p* < 0.0056/2). No substantial differences in genotype distributions between male and female cohorts were observed for other SNPs (Table S2, Figure S1).

## 4. Discussion

The outcomes of IS vary from complete recovery to persistent severe disability or death. Here, we assessed the associations of genetic variants in six genes with poststroke outcomes. Traditionally, outcomes are analyzed by dividing patients into functional and nonfunctional recovery groups based on individual mRS scores (i.e., the contrasted groups can include patients with mRS scores of 0–2 and scores of 3–6 [5] or with mRS scores of 0–3 and scores of 4–6 [25]). As the optimal grouping lacks consensus, we also subdivided patients based on the dynamics and direction of changes in their mRS scores from days 1 to 14. This grouping resulted in three different categories (positive, negative, and stable), which did not correlate directly with functional impairments after IS. The results of regression analysis showed less dependence of the proposed grouping on the age of patients and suggested that it could be an alternative to other variants in the division of patients when studying the outcomes of IS.

No associations were found between the studied SNPs and functional outcome as assessed by mRS scores. However, one SNP locus (rs66782529) was found to be nominally correlated (*p* < 0.05) with negative ∆mRS values. This SNP is 8 kb distant from the start of the *LGALS3* gene, but following formal criteria, we annotated it with this gene. *LGALS3* encodes a beta-galactoside-binding protein, galectin-3, that is involved in regulation or mediation of diverse intra- and extracellular processes, including cell adhesion, migration, differentiation, apoptosis, and inflammation [26]. Galectin-3 is ubiquitously expressed in adult humans under normal physiological conditions. Its expression was also found to be significantly and rapidly induced under disease conditions. Therefore, galectin-3 is regarded as an important biomarker, particularly of cardiovascular and cerebrovascular injuries and diseases [27,28]. This potential is associated with pro-inflammatory properties. The role of galectin-3 in mediating inflammatory responses during subacute stroke in rats has been demonstrated directly through the reversion of neuroinflammation when *LGALS3* expression was inhibited [29]. Considering the data of the Genotype-Tissue Expression (GTEx) project, which demonstrated the ability of rs66782529 genotypes to influence *LGALS3* expression in the brain, the results of our study agree with the literature. First, according to the GTEx study, the CT and TT genotypes decreased expression. Second, the maximum number of patients with a CC genotype was observed in the group with negative ΔmRS values. In that group, the decline of functional abilities was not stopped or reversed. Therefore, neuroinflammation as a factor for producing further brain damage could have been involved in these patients. Additionally, when considering the functional features of SNP rs66782529 according to the results of HaploReg [30] and RegulomeDB [31], it is interesting to note that the SNP affects *LGALS* expression influencing local chromatin state. 

Additional associations were found between the SNPs and IS. As stated above, they were evaluated because of the limitations of existing data on stroke genetics, particularly its potential specificity in populations with different ethnicities. Our analysis was performed in patients self-identifying as Russian. Both significant SNPs were located upstream of the *PTX3* gene. This encodes a pentraxin-3 protein that is an essential mediator of innate resistance to different pathogens of fungal, bacterial, and viral origin, and is involved in the regulation of inflammation, angiogenesis, tissue remodeling, and cancers [32]. As with *LGALS3*, *PTX3* expression was found to be increased under diseased conditions, and pentraxin-3 has been defined as a novel and independent prognostic marker of mortality after acute myocardial infarction (AMI) and IS [33,34]. At the same time, experiments in animal models showed that *PTX3* depletion resulted in behavioral deficits and brain damage progression in mice at a chronic phase, suggesting that pentraxin-3 might act in a time-dependent manner, intensifying inflammation early after injury and promoting tissue repair processes at a later date [35,36,37]. Here, we also did not find any correlations between the eQTL tagSNPs from *PTX3* with functional outcomes after IS but found them to relate to the risk of IS (rs62278647 and rs2316710). The risk of IS was generally correlated with the prevalence of heterozygous genotypes in our patients. Similar direction of the association was demonstrated in the study of Melegy et al. [38], who tested the association of SNP rs2305619 with AMI (SNP rs2305619 was in strong linkage disequilibrium (LD; *r*^2^ = 0.97) with our SNP rs2316710 in CEU test population from 1000 Genomes Project [15]). At the same time, no such correlations were observed in the larger study of Barbati et al. [39], who explored the influence of *PTX3* polymorphisms on the risk of AMI and pentraxin-3 plasma levels (the SNPs tested were in strong LD with our SNPs rs2316710 and rs7634847, but not with rs62278647 (*r*^2^ = 0.47–0.69), in CEU test population [15]). Given that PTX3 is a marker for prognosis after AMI and IS, and following Barbati et al. [39], we propose that the lack of correlations between *PTX3* polymorphisms and IS outcome is because the PTX3 level itself is not a causal factor for IS outcome. The results of additional functional annotations of the SNPs rs62278647 and rs2316710 seem to support this idea as both were classified as having lower ranks compared to the SNP rs66782529, and were limited to the effects on regulatory motif sequences. However, our data on the differences in genotypic associations with IS risk between male and female patients complicate the question, suggesting the possibility of sex-based specificity of *PTX3* function, including its correlation with a higher rate of IS in women [40,41]. However, this presumption requires verification in further studies.

## 5. Conclusions

Summarizing the results of the work, we can conclude that our recently developed approach in searching for genetic markers of IS was clearly effective, as it has revealed several new associations with the outcomes and risk of IS. At the same time, the study had some limitations. First was the relatively small sample size that could reduce the statistical power of our study. Second, because the analysis was restricted to local eQTLs, the potential associations of distant SNPs (trans-eQTLs) with stroke and poststroke events were not considered. Taking these factors into account will increase the abilities and power of our approach.

## Figures and Tables

**Table 1:** Genomic characteristics of the polymorphisms studied, with primer and probe sequences and PCR conditions.

rs	chr	Position (hg 19)	Alleles	Gene	Primer and Probe Sequences	Ta *, °C
rs62278647	3	157149133	A/T	*PTX3*	5’-(FAM)AAACAGACTGATACATCCA(BHQ1)-3’ 5’-(VIC)AAACAGACAGATACATCCA(BHQ2)-3’ 5’-CAGTCTCAGGTAGTATC-3’ 5’-CTGTGAAGATGGAGAA-3’	55
rs2316710	3	157150180	C/A	*PTX3*	5’-(FAM)TTTATGAACCTGGAATACA(BHQ1)-3’ 5’-(VIC)TTTATGACCCTGGAATACA(BHQ2)-3’ 5’-TGAGCTACTGAATGAA-3’ 5’-GCCTTTAAGAGAAAATATAG-3’	54
rs7634847	3	157159145	C/T	*PTX3*	5’-(FAM)TTTAATCCTTATGCCAACCA(BHQ1)-3’ 5’-(VIC)TTTAATCCTCATGCCAACC(BHQ2)-3’ 5’-ACTGCTTATCAGCTATGTA-3’ 5’-TTGGGCATTCACTATGTA-3’	58
rs1877822	17	63165077	A/G	*RGS9*	5’-(FAM)ACTCTGCTACCTCAGTT(BHQ1)-3’ 5’-(VIC)ACTCTGCTGCCTCAGT(BHQ2)-3’ 5’-GGTCTGACTGCTACATA-3’ 5’-GAGGAGGAAGAACATTTC-3’	58
rs74063268	12	13352358	T/C	*EMP1*	5’-(FAM)TCTTCAACGTGTCTCTCT(BHQ1)-3’ 5’-(VIC)TCTTCAACGCGTCTCTC(BHQ2)-3’ 5’-CAGGTAGTGCCAGAC-3’ 5’-TGCCTCATCCACAAG-3’	58
rs2569192	5	140015208	C/G	*CD14*	5’-(FAM)CTTTCTAGCAACCCTAGT(BHQ1)-3’ 5’-(VIC)CTTTCTACCAACCCTAGT(BHQ2)-3’ 5’-CAGCTTGATTCAACAA-3’ 5’-AGGGACTTGTCTTTG-3’	55
rs66782529	14	55587867	C/T	*LGALS3*	5’-(FAM)AGTTATTTGACTCTCTGTGAC(BHQ1)-3’ 5’-(VIC)AGTTATTTGATTCTCTGTGACT(BHQ2)-3’ 5’-CTGACACTTACCAGTTG-3’ 5’-GCTCACAACAAACCTATA-3’	58
rs1009977	14	55603002	T/G	*LGALS3*	5’-(FAM) ACTGCCAAATATTTTATTTG(BHQ1)-3’ 5’-(VIC) CTGCCAAAGATTTTATTTG(BHQ2)-3’ 5’-GCATCTTAGACCCAAA-3’ 5’-GCTGTTGGAGCTAAG-3’	55
rs1491961	3	46250348	T/C	*CCR1*	5’-(FAM)TGGCACCCTATGCCC(BHQ1)-3’ 5’-(VIC)TGGCACCCCATGCC(BHQ2)-3’ 5’-CTGACATGGGTGCTCAC-3’ 5’-CCTTCATGCTGGAGGTTC-3’	62

* Ta—annealing temperature.

**Table 2:** Frequency of genotypes in the groups of patients from Studies 1, 2 and 3.

SNP	Gene	Genotype	Study 1 *		*p*	Study 2 *		*p*	Study 3 *			*p*
			mRS 0–2	mRS 3–6		mRS 0–3	mRS 4–6		ΔmRS > 0	ΔmRS < 0	ΔmRS = 0	
rs62278647	*PTX3*	TT	0.20 (23)	0.25 (53)	0.2375	0.22 (39)	0.24 (37)	0.6600	0.23 (35)	0.22 (15)	0.24 (26)	0.5626
		AT	0.67 (78)	0.58 (123)		0.63 (110)	0.59 (91)		0.64 (98)	0.55 (37)	0.61 (66)	
		AA	0.13 (15)	0.17 (37)		0.14 (25)	0.17 (27)		0.14 (21)	0.22 (15)	0.15 (16)	
rs2316710	*PTX3*	CC	0.10 (12)	0.16 (34)	0.3577	0.12 (21)	0.16 (25)	0.3881	0.11 (17)	0.21 (14)	0.14 (15)	0.3980
		CA	0.59 (69)	0.55 (116)		0.56 (97)	0.57 (88)		0.59 (91)	0.52 (34)	0.56 (60)	
		AA	0.30 (35)	0.29 (62)		0.32 (56)	0.27 (41)		0.30 (46)	0.27 (18)	0.31 (33)	
rs7634847	*PTX3*	CC	0.41 (48)	0.40 (85)	0.5256	0.42 (73)	0.39 (60)	0.5311	0.39 (60)	0.42 (28)	0.42 (45)	0.4598
		CT	0.51 (59)	0.48 (103)		0.49 (86)	0.49 (76)		0.53 (82)	0.43 (29)	0.47 (51)	
		TT	0.08 (9)	0.12 (25)		0.09 (15)	0.12 (19)		0.08 (12)	0.15 (10)	0.11 (12)	
rs1877822	*RGS9*	AA	0.65 (75)	0.67 (144)	0.6443	0.65 (113)	0.68 (106)	0.7086	0.65 (100)	0.61 (41)	0.72 (78)	0.3930
		GA	0.32 (37)	0.31 (66)		0.33 (57)	0.30 (46)		0.33 (51)	0.34 (23)	0.27 (29)	
		GG	0.03 (4)	0.02 (4)		0.03 (5)	0.02 (3)		0.03 (4)	0.04 (3)	0.01 (1)	
rs74063268	*EMP1*	TT	0.87 (101)	0.83 (178)	0.6348	0.85 (149)	0.84 (130)	0.9496	0.85 (131)	0.84 (56)	0.85 (92)	0.7112
		CT	0.12 (14)	0.15 (33)		0.14 (24)	0.15 (23)		0.14 (21)	0.15 (10)	0.15 (16)	
		CC	0.01 (1)	0.01 (3)		0.01 (2)	0.01 (2)		0.02 (3)	0.01 (1)	0.00 (0)	
rs2569192	*CD14*	CC	0.45 (52)	0.47 (99)	0.5947	0.45 (77)	0.48 (74)	0.5365	0.45 (69)	0.44 (29)	0.50 (53)	0.8523
		CG	0.50 (58)	0.45 (96)		0.50 (86)	0.44 (68)		0.49 (75)	0.47 (31)	0.45 (48)	
		GG	0.05 (6)	0.08 (1)6		0.06 (10)	0.08 (12)		0.06 (10)	0.09 (6)	0.06 (6)	
rs66782529	*LGALS3*	CC	0.46 (53)	0.51 (109)	0.6104	0.44 (77)	0.55 (85)	0.1469	0.45 (70)	0.61 (41)	0.47 (51)	0.0488
		TC	0.45 (52)	0.39 (84)		0.46 (80)	0.36 (56)		0.44 (67)	0.27 (18)	0.47 (51)	
		TT	0.09 (11)	0.09 (20)		0.10 (17)	0.09 (14)		0.11 (17)	0.12 (8)	0.06 (6)	
rs1009977	*LGALS3*	TT	0.40 (46)	0.36 (78)	0.4536	0.33 (58)	0.43 (66)	0.2069	0.35 (55)	0.46 (31)	0.35 (38)	0.5467
		GT	0.42 (49)	0.49 (105)		0.50 (88)	0.43 (66)		0.48 (74)	0.39 (26)	0.50 (54)	
		GG	0.18 (21)	0.14 (31)		0.17 (29)	0.15 (23)		0.17 (26)	0.15 (10)	0.15 (16)	
rs1491961	*CCR1*	CC	0.41 (48)	0.46 (98)	0.3249	0.42 (73)	0.47 (73)	0.0773	0.44 (68)	0.46 (31)	0.44 (47)	0.9413
		CT	0.51 (59)	0.43 (92)		0.51 (89)	0.40 (62)		0.47 (73)	0.42 (28)	0.46 (50)	
		TT	0.08 (9)	0.11 (24)		0.07 (13)	0.13 (20)		0.09 (14)	0.12 (8)	0.10 (11)	

* Numbers in brackets are the numbers of individuals with particular genotypes.

**Table 3:** Frequency of genotypes in the groups of patients and controls.

SNP	Gene	Genotypes	Patients *	Controls *	Chi-Square **	*p* **
rs62278647	*PTX3*	TT	0.23 (76)	0.28 (63)	20.9166	0.000029 ^†^
		AT	0.61 (201)	0.42 (94)		
		AA	0.16 (53)	0.29 (65)		
rs2316710	*PTX3*	CC	0.14 (47)	0.25 (56)	11.99	0.0025 ^†^
		CA	0.56 (185)	0.45 (99)		
		AA	0.29 (97)	0.30 (67)		
rs7634847	*PTX3*	CC	0.40 (133)	0.41 (92)	5.2685	0.0718
		CT	0.49 (162)	0.42 (93)		
		TT	0.11 (35)	0.17 (37)		
rs1877822	*RGS9*	AA	0.66 (220)	0.63 (140)	1.08678	0.5808
		GA	0.31 (103)	0.33 (74)		
		GG	0.02 (8)	0.04 (8)		
rs74063268	*EMP1*	TT	0.85 (280)	0.82 (181)	2.22535	0.3287
		CT	0.14 (47)	0.18 (40)		
		CC	0.01 (4)	0.00 (1)		
rs2569192	*CD14*	CC	0.46 (151)	0.50 (110)	0.698198	0.7053
		CG	0.47 (155)	0.45 (99)		
		GG	0.07 (22)	0.06 (13)		
rs66782529	*LGALS3*	CC	0.49 (162)	0.47 (104)	0.474517	0.7888
		TC	0.41 (136)	0.44 (98)		
		TT	0.10 (32)	0.09 (20)		
rs1009977	*LGALS3*	TT	0.37 (124)	0.38 (85)	0.26155	0.8774
		GT	0.47 (154)	0.47 (105)		
		GG	0.16 (53)	0.14 (32)		
rs1491961	*CCR1*	CC	0.44 (146)	0.52 (115)	4.97237	0.0832
		CT	0.46 (152)	0.42 (92)		
		TT	0.10 (33)	0.06 (13)		

* Numbers in brackets are the numbers of individuals with particular genotypes; ** Genotype distribution in groups was compared by genotypic test; ^†^ Significant *p*-values.

## Data Availability

The data presented in this study are available in Supplementary Materials here.

## Supplementary Materials

The following are available online at https://www.mdpi.com/article/10.3390/genes12121938/s1, Figure S1: Frequency of genotypes in patients and controls among males and females, Table S1: The raw genotype and phenotype data analyzed in the study, Table S2: Frequency of genotypes in patients and controls among males and females.

## References

[1] Campbell B.C.V., De Silva D.A., MacLeod M.R., Coutts S.B., Schwamm L.H., Davis S.M., Donnan G.A. (2019). Ischaemic stroke. Nat. Rev. Dis. Prim..

[2] Chauhan G., Debette S. (2016). Genetic Risk Factors for Ischemic and Hemorrhagic Stroke. Curr. Cardiol. Rep..

[3] Malik R., Chauhan G., Traylor M., Sargurupremraj M., Okada Y., Mishra A., Rutten-Jacobs L., Giese A.-K., van der Laan S.W., AFGen Consortium (2018). Multiancestry genome-wide association study of 520,000 subjects identifies 32 loci associated with stroke and stroke subtypes. Nat. Genet..

[4] Lindgren A., Maguire J. (2016). Stroke Recovery Genetics. Stroke.

[5] Söderholm M., Pedersen A., Lorentzen E., Stanne T.M., Bevan S., Olsson M., Cole J.W., Fernandez-Cadenas I., Hankey G.J., Jimenez-Conde J. (2019). Genome-wide association meta-analysis of functional outcome after ischemic stroke. Neurology.

[6] Mola-Caminal M., Carrera C., Soriano-Tárraga C., Giralt-Steinhauer E., Díaz-Navarro R.M., Tur S., Jiménez C., Medina-Dols A., Cullell N., Torres-Aguila N.P. (2019). PATJ Low Frequency Variants Are Associated With Worse Ischemic Stroke Functional Outcome. Circ. Res..

[7] Ibanez L., Heitsch L., Carrera C., Farias F.H.G., Dhar R., Budde J., Bergmann K., Bradley J., Harari O., Phuah C.-L. (2020). Multi-ancestry genetic study in 5,876 patients identifies an association between excitotoxic genes and early outcomes after acute ischemic stroke. medRxiv Prepr. Serv. Heal. Sci..

[8] Gallagher M.D., Chen-Plotkin A.S. (2018). The Post-GWAS Era: From Association to Function. Am. J. Hum. Genet..

[9] Nicholls H.L., John C.R., Watson D., Munroe P.B., Barnes M.R., Cabrera C.P. (2020). Reaching the End-Game for GWAS: Machine Learning Approaches for the Prioritization of Complex Disease Loci. Front. Genet..

[10] Zheng Q., Ma Y., Chen S., Che Q., Chen D. (2020). The Integrated Landscape of Biological Candidate Causal Genes in Coronary Artery Disease. Front. Genet..

[11] Dergunova L.V., Filippenkov I.B., Stavchansky V.V., Denisova A.E., Yuzhakov V.V., Mozerov S.A., Gubsky L.V., Limborska S.A. (2018). Genome-wide transcriptome analysis using RNA-Seq reveals a large number of differentially expressed genes in a transient MCAO rat model. BMC Genom..

[12] Ma R., Xie Q., Li Y., Chen Z., Ren M., Chen H., Li H., Li J., Wang J. (2020). Animal models of cerebral ischemia: A review. Biomed. Pharmacother..

[13] Wang Y., Cai Y. (2016). Obtaining Human Ischemic Stroke Gene Expression Biomarkers from Animal Models: A Cross-species Validation Study. Sci. Rep..

[14] Brubaker D.K., Lauffenburger D.A. (2020). Translating preclinical models to humans. Science.

[15] Khvorykh G., Khrunin A., Filippenkov I., Stavchansky V., Dergunova L., Limborska S. (2021). A Workflow for Selection of Single Nucleotide Polymorphic Markers for Studying of Genetics of Ischemic Stroke Outcomes. Genes.

[16] Pasterkamp G., Van Der Laan S.W., Haitjema S., Asl H.F., Siemelink M.A., Bezemer T., Van Setten J., Dichgans M., Malik R., Worrall B.B. (2016). Human Validation of Genes Associated With a Murine Atherosclerotic Phenotype. Arter. Thromb. Vasc. Biol..

[17] Shetova I.M., Timofeev D.I., A Shamalov N., A Bondarenko E., A Slominskiĭ P., A Limborskaia S., Skvortsova V.I. (2012). The association between the DNA marker rs1842993 and risk for cardioembolic stroke in the Slavic population. Zhurnal Nevrol. i psikhiatrii im. S.S. Korsakova.

[18] Adams H.P., Bendixen B.H., Kappelle L.J., Biller J., Love B.B., Gordon D.L., Marsh E.E. (1993). Classification of subtype of acute ischemic stroke. Definitions for use in a multicenter clinical trial. TOAST. Trial of Org 10172 in Acute Stroke Treatment. Stroke.

[19] Brott T., Adams H.P., Olinger C.P., Marler J.R., Barsan W.G., Biller J., Spilker J., Holleran R., Eberle R., Hertzberg V. (1989). Measurements of acute cerebral infarction: A clinical examination scale. Stroke.

[20] van Swieten J.C., Koudstaal P.J., Visser M.C., Schouten H.J., van Gijn J. (1988). Interobserver agreement for the assessment of handicap in stroke patients. Stroke.

[21] Milligan B.G., Hoelzel A. (1998). Total DNA isolation. Molecular Genetic Analysis of Populations.

[22] Roy-O’Reilly M.M., McCullough M.L.D. (2018). Age and Sex Are Critical Factors in Ischemic Stroke Pathology. Endocrinology.

[23] Wang L., Zhang W., Li Q. (2020). AssocTests: An R Package for Genetic Association Studies. J. Stat. Softw..

[24] Barrett J., Fry B., Maller J., Daly M.J. (2004). Haploview: Analysis and visualization of LD and haplotype maps. Bioinformatics.

[25] Kongsawasdi S., Klaphajone J., Wivatvongvana P., Watcharasaksilp K. (2019). Prognostic Factors of Functional Outcome Assessed by Using the Modified Rankin Scale in Subacute Ischemic Stroke. J. Clin. Med. Res..

[26] Sciacchitano S., Lavra L., Morgante A., Ulivieri A., Magi F., De Francesco G.P., Bellotti C., Salehi L.B., Ricci A. (2018). Galectin-3: One Molecule for an Alphabet of Diseases, from A to Z. Int. J. Mol. Sci..

[27] Gao Z., Liu Z., Wang R., Zheng Y., Li H., Yang L. (2020). Galectin-3 Is a Potential Mediator for Atherosclerosis. J. Immunol. Res..

[28] Shen Y.-F., Yu W.-H., Dong X.-Q., Du Q., Yang D.-B., Wu G.-Q., Zhang Z.-Y., Wang H., Jiang L. (2016). The change of plasma galectin-3 concentrations after traumatic brain injury. Clin. Chim. Acta.

[29] Wang Y., He S., Liu X., Li Z., Zhu L., Xiao G., Du X., Du H., Zhang W., Zhang Y. (2021). Galectin-3 Mediated Inflammatory Response Contributes to Neurological Recovery by QiShenYiQi in Subacute Stroke Model. Front. Pharmacol..

[30] Ward L.D., Kellis M. (2015). HaploReg v4: Systematic mining of putative causal variants, cell types, regulators and target genes for human complex traits and disease. Nucleic Acids Res..

[31] Boyle A.P., Hong E.L., Hariharan M., Cheng Y., Schaub M.A., Kasowski M., Karczewski K.J., Park J., Hitz B.C., Weng S. (2012). Annotation of functional variation in personal genomes using RegulomeDB. Genome Res..

[32] Bottazzi B., Garlanda C., Teixeira M.M. (2019). Editorial: The Role of Pentraxins: From Inflammation, Tissue Repair and Immunity to Biomarkers. Front. Immunol..

[33] Latini R., Maggioni A.P., Peri G., Gonzini L., Lucci D., Mocarelli P., Vago L., Pasqualini F., Signorini S., Soldateschi D. (2004). Prognostic Significance of the Long Pentraxin PTX3 in Acute Myocardial Infarction. Circulation.

[34] Ryu W.-S., Kim C.K., Kim B.J., Kim C., Lee S.-H., Yoon B.-W. (2012). Pentraxin 3: A novel and independent prognostic marker in ischemic stroke. Atherosclerosis.

[35] Rodriguez-Grande B., Varghese L., Molina-Holgado F., Rajkovic O., Garlanda C., Denes A., Pinteaux E. (2015). Pentraxin 3 mediates neurogenesis and angiogenesis after cerebral ischaemia. J. Neuroinflammation.

[36] Oggioni M., Mercurio D., Minuta D., Fumagalli S., Popiolek-Barczyk K., Sironi M., Ciechanowska A., Ippati S., De Blasio D., Perego C. (2021). Long pentraxin PTX3 is upregulated systemically and centrally after experimental neurotrauma, but its depletion leaves unaltered sensorimotor deficits or histopathology. Sci. Rep..

[37] Rodriguez-Grande B., Swana M., Nguyen L., Englezou P., Maysami S., Allan S., Rothwell N.J., Garlanda C., Denes A., Pinteaux E. (2013). The Acute-Phase Protein PTX3 is an Essential Mediator of Glial Scar Formation and Resolution of Brain Edema after Ischemic Injury. Br. J. Pharmacol..

[38] El Melegy E.K., Badr E.A., ElKersh A.M., EL Shafey W.H., Fareed W.A. (2016). Pentraxin 3 genotyping in relation to serum levels of pentraxin 3 in patients with acute ST-segment elevation myocardial infarction. Clin. Trials Regul. Sci. Cardiol..

[39] Barbati E., Specchia C., Villella M., Rossi M.L., Barlera S., Bottazzi B., Crociati L., D’Arienzo C., Fanelli R., Garlanda C. (2012). Influence of Pentraxin 3 (PTX3) Genetic Variants on Myocardial Infarction Risk and PTX3 Plasma Levels. PLoS ONE.

[40] Seshadri S., Beiser A., Kelly-Hayes M., Kase C.S., Au R., Kannel W.B., Wolf P.A. (2006). The Lifetime Risk of Stroke. Stroke.

[41] Benjamin E.J., Blaha M.J., Chiuve S.E., Cushman M., Das S.R., Deo R., de Ferranti S.D., Floyd J., Fornage M., Gillespie C. (2017). Heart disease and stroke statistics—2017 update: A report from the American heart association. Circulation.

