# Supplementary material for: Examination of Genetic Variants Revealed from a Rat Model of Brain Ischemia in Patients with Ischemic Stroke: A Pilot Study
Source: Genes (Basel). 2021 Nov 30;12(12):1938. doi: 10.3390/genes12121938 (PMC8701352; doi:10.3390/genes12121938)
Supplement: Supplementary file 1 [file genes-12-01938-s001.zip › genes-1461615-supplementary/Table S2.pdf]

genes

MDPI

Table S2. Frequency of genotypes in patients and controls among men and women.

SNP	Gene	Genotype	Men*		P	Women*		P
			Controls	Patients		Controls	Patients	
rs62278647	*PTX3*	TT	0.28 (31)	0.24 (39)	0.1220	0.28 (32)	0.22 (37)	0.000057
		AT	0.46 (50)	0.58 (96)		0.39 (44)	0.64 (105)	
		AA	0.20 (28)	0.18 (30)		0.33 (37)	0.14 (23)	
rs2316710	*PTX3*	CC	0.21 (23)	0.16 (27)	0.5637	0.29 (33)	0.12 (20)	0.00077
		CA	0.48 (52)	0.53 (87)		042 (47)	0.59 (98)	
		AA	0.31 (34)	0.30 (50)		0.29 (33)	0.28 (47)	
rs7634847	*PTX3*	CC	0.41 (45)	0.37 (61)	0.5257	0.42 (47)	0.44 (72)	0.0616
		CT	0.44 (48)	0.51 (84)		0.40 (45)	0.47 (78)	
		TT	0.15 (16)	0.12 (20)		0.19 (21)	0.09 (15)	
rs1877822	*RGS9*	AA	0.56 (61)	0.67 (112)	0.1332	0.70 (79)	0.65 (108)	0.7047
		GA	0.39 (43)	0.30 (50)		0.27 (31)	0.32 (53)	
		GG	0.05 (5)	0.02 (4)		0.03 (3)	0.02 (4)	
rs74063268	*EMP1*	TT	0.82 (89)	0.86 (142)	0.3654	0.81 (92)	0.84 (138)	0.1578
		CT	0.17 (19)	0.14 (24)		0.19 (21)	0.14 (23)	
		CC	0.01 (1)	0.00 (0)		0.00 (0)	0.02 (4)	
rs2569192	*CD14*	CC	0.47 (51)	0.44 (72)	0.7370	0.52 (59)	0.48 (79)	0.8151
		CG	0.50 (54)	0.51 (84)		0.40 (45)	0.44 (71)	
		GG	0.04 (4)	0.05 (9)		0.08 (9)	0.08 (13)	
rs66782529	*LGALS3*	CC	0.45 (49)	0.47 (77)	0.8661	0.49 (55)	0.52 (85)	0.4748
		TC	0.44 (48)	0.44 (73)		0.44 (50)	0.38 (63)	
		TT	0.11 (12)	0.09 (15)		0.07 (8)	0.10 (17)	
rs1009977	*LGALS3*	TT	0.34 (37)	0.35 (58)	0.8978	0.42 (48)	0.40 (66)	0.5016
		GT	0.50 (54)	0.51 (84)		0.45 (51)	0.42 (70)	
		GG	0.17 (18)	0.14 (24)		0.12 (14)	0.18 (29)	
rs1491961	*CCR1*	CC	0.52 (57)	0.41 (68)	0.1042	0.52 (58)	0.47 (78)	0.5903
		CT	0.42 (46)	0.48 (80)		0.41 (46)	0.44 (72)	
		TT	0.06 (6)	0.11 (18)		0.06 (7)	0.09 (15)	

*Numbers in squares are the numbers of individuals with particular genotypes.
