# Supplementary figures and images for: Examination of Genetic Variants Revealed from a Rat Model of Brain Ischemia in Patients with Ischemic Stroke: A Pilot Study
Source: Genes (Basel). 2021 Nov 30;12(12):1938. doi: 10.3390/genes12121938 (PMC8701352; doi:10.3390/genes12121938)

rs2316710\_A

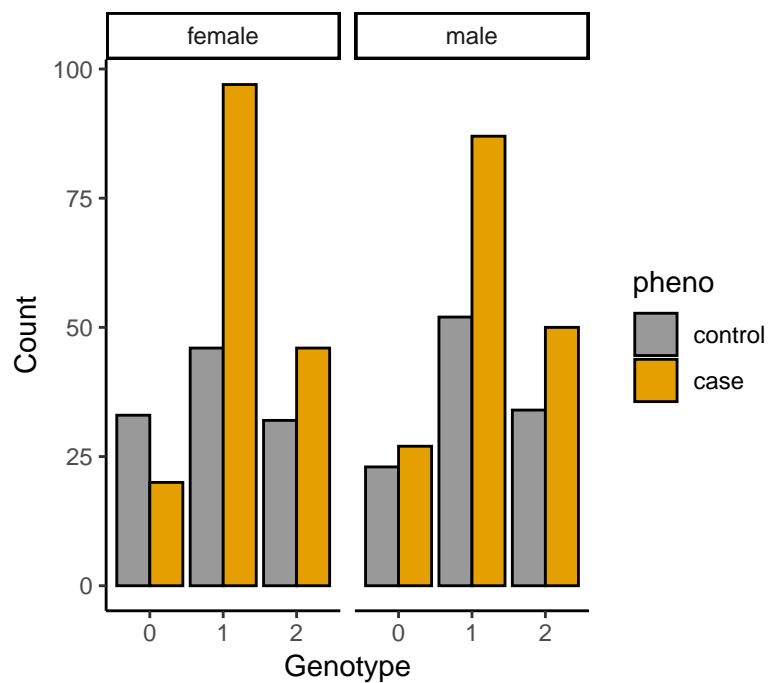

rs1877822\_A

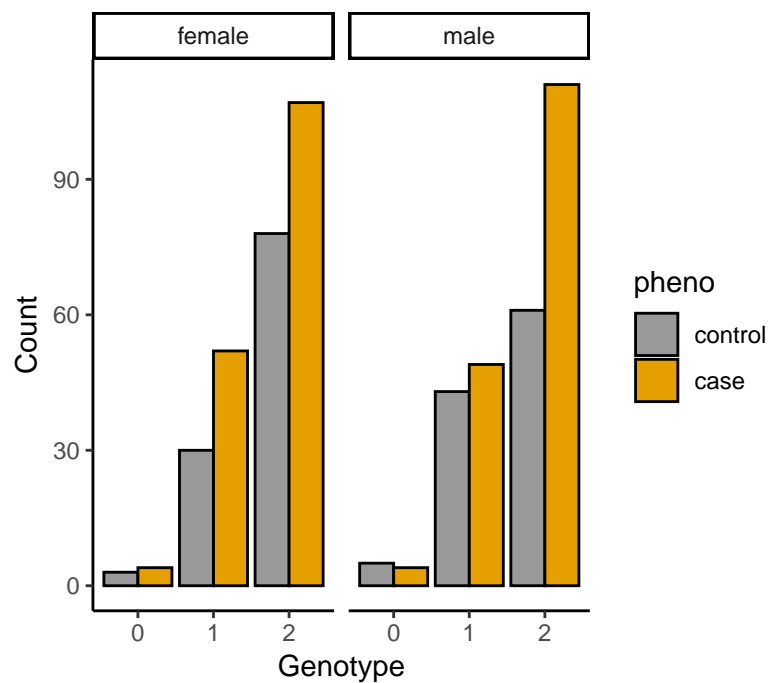

rs7634847\_C

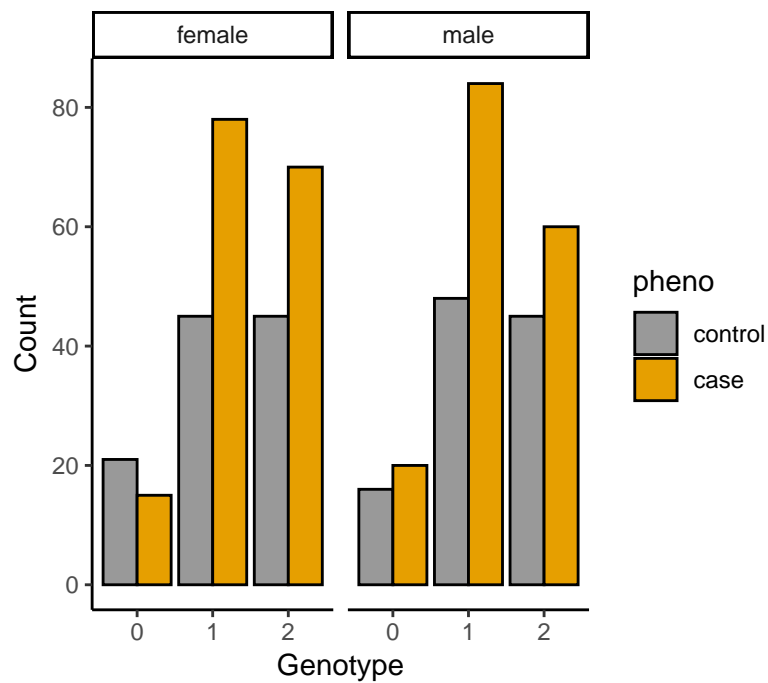

rs74063268\_T

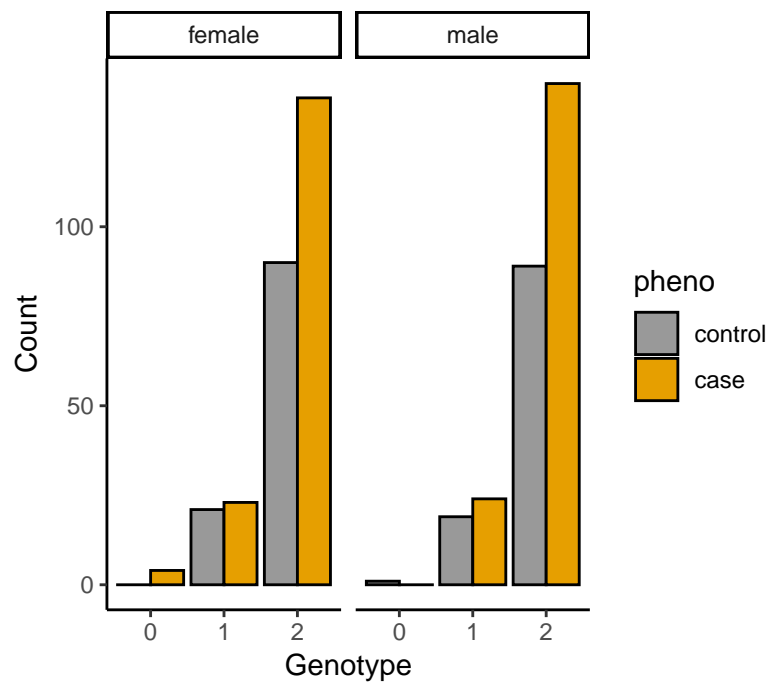

rs62278647\_T

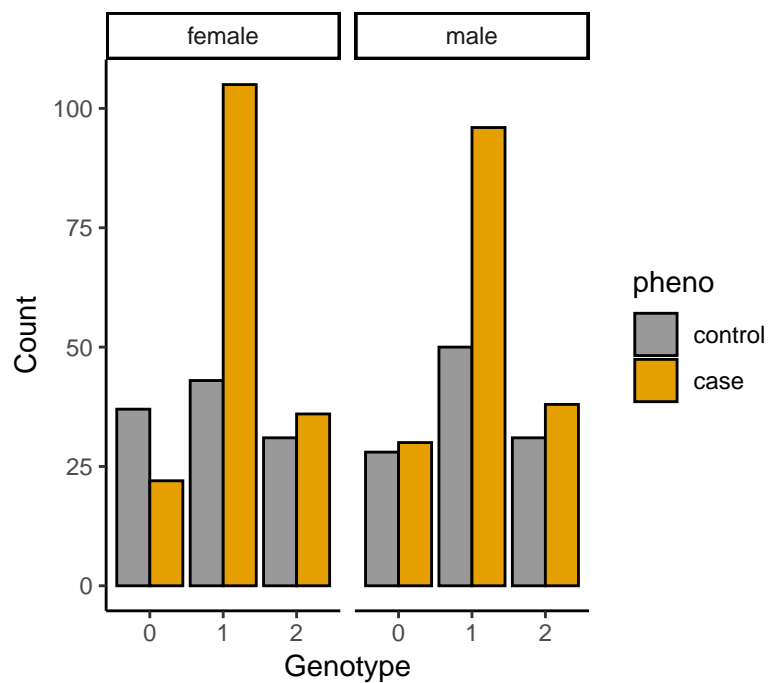

rs2569192\_C

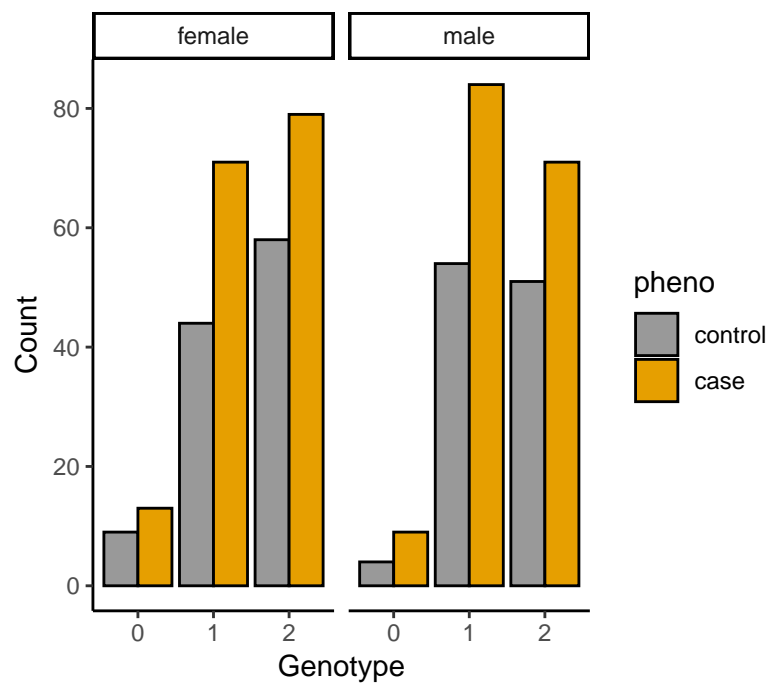

rs66782529\_C

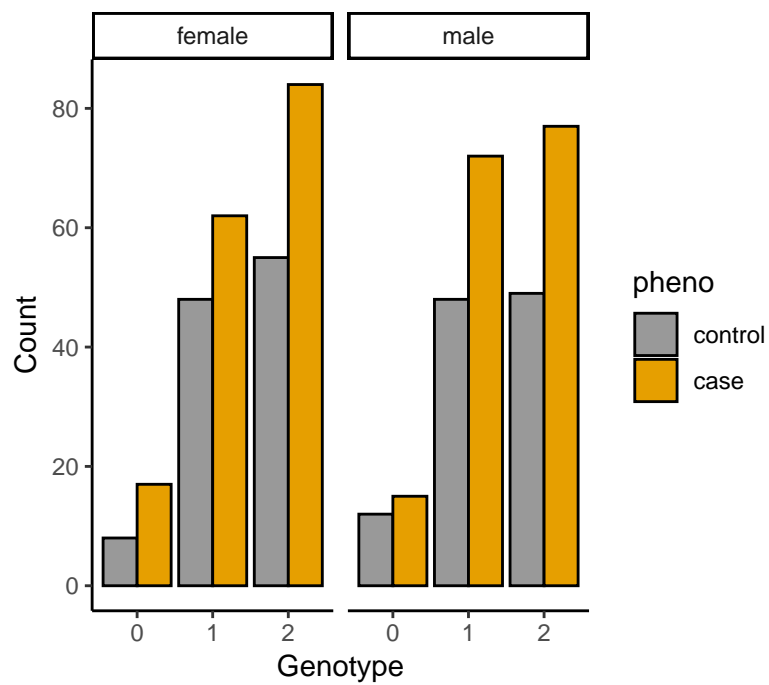

rs1491961\_C

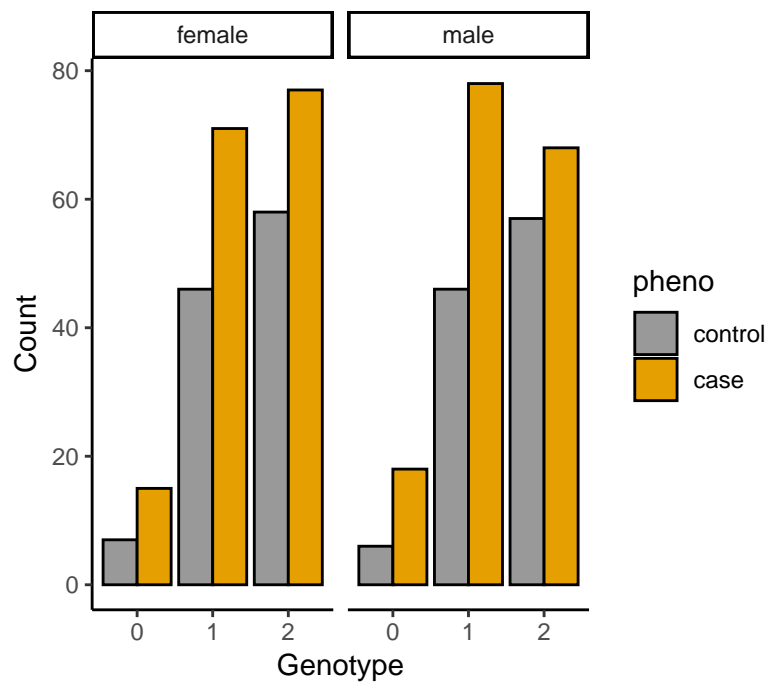

rs1009977\_T

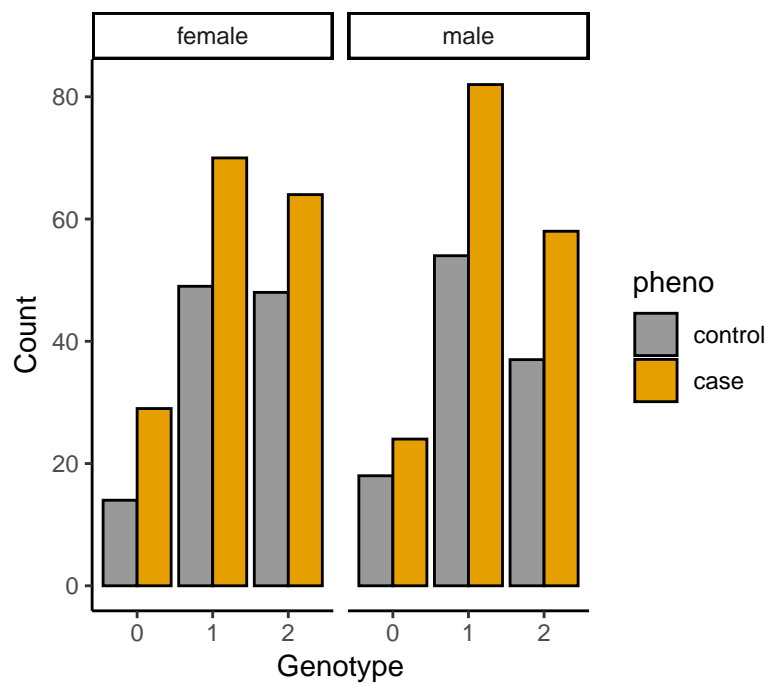

Supplement: Supplementary file 1 [file genes-12-01938-s001.zip › genes-1461615-supplementary/Figure S1.pdf]
